# Adrenomedullin in plasma of surgical ICU-patients with sepsis - a pilot study

**DOI:** 10.1186/2197-425X-3-S1-A302

**Published:** 2015-10-01

**Authors:** T-P Simon, S Doemming, A Humbs, L Martin, C Bruells, O Hartmann, J Struck, A Bergmann, G Marx, T Schuerholz

**Affiliations:** University Hospital RWTH Aachen, Department of Intensive Care and Intermediate Care, Aachen, Germany; Sphingotec GmbH, Hennigsdorf, Germany

## Introduction

Synthesis and liberation of adrenomedullin (ADM) are found in different tissues and endothelial cells and ADM is induced by hypoxia, oxidative stress, and pro-inflammatory cytokines [1]. The prognostic value of ADM is described in general patient populations, in medical patients and in particular recently in patients with suspected sepsis in the emergency department [2, 3]. In surgical patients, the obstacle is to differentiate between postoperative inflammation and sepsis and to assess the patients' prognosis.

## Objectives

To evaluate the value of measuring ADM in surgical ICU-patients with sepsis, severe sepsis or septic shock.

## Methods

In a prospective, observational clinical trial, we included 42 consecutive ICU patients after major surgery with clinical signs of sepsis according to the ACCP/SCCM definitions and 14 patients admitted routinely to the ICU after major surgery. Plasma samples to determine ADM were drawn within 16 hours after diagnosis of sepsis or routine admission. Laboratory and clinical parameters and 28-day and 90-day mortality were recorded. Values are expressed as median and interquartile ranges (IQR), or counts and percentages as appropriate. Group comparisons of continuous variables were performed using Kruskal-Wallis test. Biomarker data were log-transformed. Spearman rank-order correlation was applied to continuous variables. A p-value of < 0.05 was considered significant.

## Results

Patients (66% male) were 70 (IQR 60.5-77) years old and had a body mass index of 26.2 (IQR 24.2-29.4) kg/m². Lengths of stay (LOS) in ICU was 3 (IQR 1-12) days and in hospital 16.5 (IQR 8-21) days. Of the 42 consecutive ICU patients, eight patients had sepsis, 19 developed severe sepsis and 15 suffered from septic shock. ADM increased with severity (p < 0.0001, table 1).

**Table 1:** ADM concentrations in all groups

Subgroup	n	Median (pg/mL)	IQR (pg/mL)
Control	14	16.2	11.8 - 20.0
Sepsis	8	25.8	20.3 - 40.2
Severe sepsis	19	84.2	42.7 - 118.5
Septic shock	15	119.7	83.8 - 172.6

Adrenomedullin (ADM) levels in control, sepsis, severe sepsis and septic shock patients (p < 0.0001). Post-hoc comparisons show significant differences between controls and severe sepsis/septic shock, as well as sepsis and severe sepsis/septic shock.

**Figure 1:**
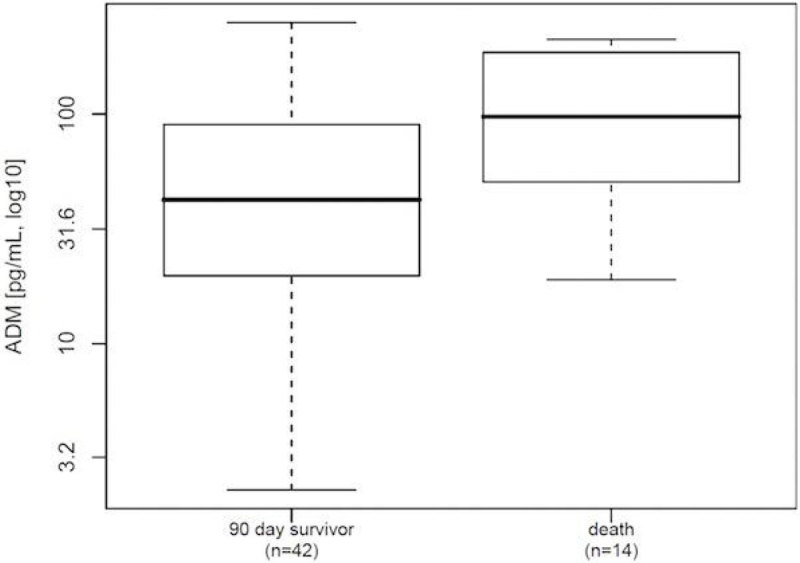
**ADM by 90 day outcome (p = 0.019).**

**Figure 2:**
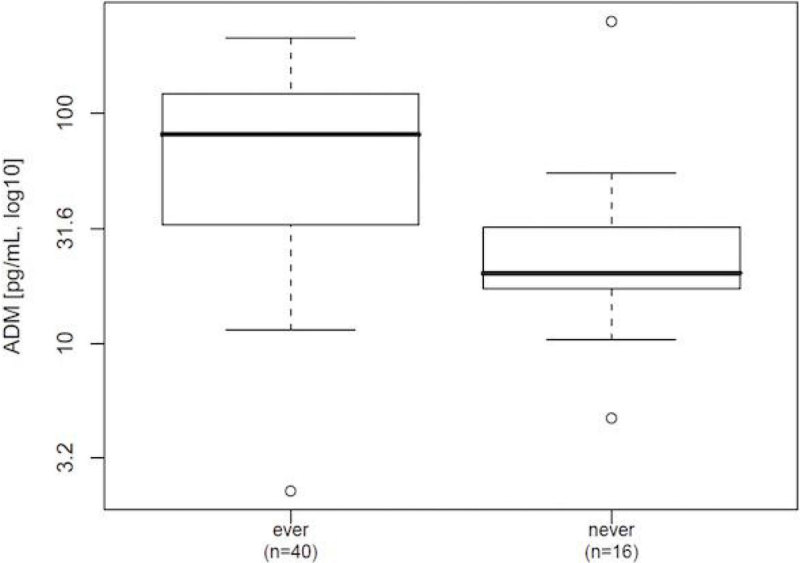
**Vasopressor use during stay (p = 0.001).**

Higher ADM concentrations were associated with poor 90 day outcome (p = 0.019, figure 1) and more frequent vasopressor usage (p = 0.001, figure 2)

## Conclusions

Adrenomedullin levels are increased according to the severity of sepsis in surgical ICU patients. Higher levels of adrenomedullin predict the need of vasopressor and survival. Thus, adrenomedullin may be a useful additional parameter in surgical patients with suspected sepsis.

## Grant Acknowledgment

This study was supported by a restricted grant of Sphingotec, Henningsdorf, Germany.
